# Associations and Similarities Between Breast Cancer and Vulvar Cancer: A Comprehensive Literature Review

**DOI:** 10.3390/medicina62101914

**Published:** 2026-10-03

**Authors:** Sinziana Ionescu, Mirela Gherghe, Silviu Voinea, Silvia Alexandra Bocai, Augustin Marian Marincas, Daniela Elena Mihaila, Ariana Neicu, Madalina Radu, Adina Ene, Robert Rosca, Dan Luca, Denisa Rauoa Barakat, Laurentiu Simion

**Affiliations:** 1 Surgery Department, “Carol Davila” University of Medicine and Pharmacy, 050474 Bucharest, Romania; sinzianaionescu30@gmail.com (S.I.);; 2 General Surgery and Surgical Oncology Clinic I, Bucharest Institute of Oncology “Prof. Dr. Al. Trestioreanu”, 022328 Bucharest, Romania; 3 Nuclear Medicine Department, Bucharest Institute of Oncology “Prof. Dr. Al. Trestioreanu”, 022328 Bucharest, Romania; mirela.gherghe@umfcd.ro; 4 Nuclear Medicine Department, “Carol Davila” University of Medicine and Pharmacy, 050474 Bucharest, Romania; 5 General Surgery and Surgical Oncology Clinic II, Bucharest Institute of Oncology “Prof. Dr. Al. Trestioreanu”, 022328 Bucharest, Romania; 6 “Carol Davila” University of Medicine and Pharmacy, 050474 Bucharest, Romania; 7 Radiotherapy Department, Pitesti Oncofort Hospital, 110283 Pitesti, Romania; 8 General Surgery Department, “Agrippa Ionescu” Emergency Hospital, 011356 Bucharest, Romania; 9 Pathology Department, Bucharest Institute of Oncology “Prof. Dr. Al. Trestioreanu”, 022328 Bucharest, Romania

**Keywords:** breast cancer, vulvar cancer, BRCA, HPV, gynaecologic cancer, surgical oncology, AMGT, genetic overlap, biological root, similarities across cancers

## Abstract

*Introduction*: Breast cancer and vulvar cancer are two distinct types of gynecological tumors with different clinical presentations; nonetheless, they share notable molecular, genetic, and epidemiological characteristics. This narrative literature review aims to synthesize existing data to elucidate the shared risk factors, genetic modifications, and pathophysiological mechanisms linking these two malignancies. *Material and methods*: A comprehensive search of the PubMed, Scopus, and Oxford Journals Library databases was performed to locate peer-reviewed research published up to 2026. The search was performed according to the following terms: “Associations and similarities between breast cancer and vulvar cancer”. Studies assessing epidemiological linkages, common genetic associations, or particular types of malignancies such as “Primary vulvar adenocarcinoma of mammary gland type (AMGT)” were included for qualitative synthesis. *Results*: The search for “Associations and similarities between breast cancer and vulvar cancer”, “breast metastases from vulvar cancer” and “vulvar metastases from breast cancer” returned various results, which were synthetized into seven main subjects: (1) breast-like tissue in the vulva, a topic which develops the themes of ectopic breast tissue, mammary-like glands and shared diseases; (2) mammary-like adenocarcinoma of the vulva (a rare form with relevant diagnostic markers); (3) metastatic and overlapping origins; (4) genetic and hormonal connections, comprising BRCA mutations and hormonal sensitivity; (5) the role of viruses such as HPV in the etiology of both cancers, a highly controversial subject in the contemporary scientific literature; (6) recurrence patterns; and (7) prognostic factors. *Conclusions*: Tumors of the breast and vulva have notable biochemical and genetic interrelations that surpass their distinct classifications. Recognizing these associations is essential for developing targeted screening techniques, identifying high-risk people, and exploring dual-efficacy therapeutic options.

## 1. Introduction

Breast cancer and vulvar cancer share surprising biological, genetic, and clinical overlaps that challenge traditional views of these distinct anatomical diseases.

The partial involution of ectodermal mammary ridges results in ectopic breast tissue (EBT) along the embryonic milk line, an uncommon congenital defect. Orthotopic thoracic presentations contrast sharply with exceptional caudal localizations in the vulva that can undergo identical hormonal and carcinogenic transformations, as explained by Loo [1]. While vulvar cancer is an uncommon gynecological disease that mostly affects older postmenopausal women, breast cancer is a high-incidence, systemic-screening cancer, as shown by Tan [2], Papatheodorou [3] and Zhang [4]. Breast cancer is the most prevalent neoplasm among females, impacting one in nine globally. In contrast, vulvar cancer is uncommon, comprising <1% of female malignancies. Only 5–8% of vulvar malignancies are metastatic. Breast cancer may spread to the vulva or originate from ectopic breast tissue, as explained by Berghe [5].

At the anatomical level, the existence of mammary-like glands and aberrant breast tissue in the vulva may lead to primary breast-like carcinomas in that area, creating a distinct histogenetic connection. This anatomical connection is further shown in their histological resemblances, as both malignancies may have analogous cellular configurations, including triple-negative and basal-like characteristics. Moreover, neoplasms in both sites often exhibit the presence of estrogen and progesterone receptors, indicating common hormonal pathways that affect tumor dynamics. In addition to these physical characteristics, genetic predisposition to cancer is crucial, since germline mutations in the BRCA1 and BRCA2 genes substantially increase the likelihood of developing breast and certain gynecological cancers. Although human papillomavirus (HPV) and persistent inflammatory agents are recognized as significant contributors to vulvar cancer development, ongoing studies are exploring possible viral and environmental microenvironments that might mirror oncogenic mechanisms in breast tissue. These common etiological and molecular changes eventually result in similar metastatic patterns and unique clinical manifestations. Thus, acknowledging these intersections has significant ramifications for diagnosis and therapy, allowing physicians to modify effective breast cancer protocols—like PARP inhibitors for BRCA-mutated tumors—for targeted treatments in vulvar cancer.

Contemporary oncology studies mostly regard breast cancer and vulvar cancer as entirely distinct clinical conditions. Although research investigates individual connections such as BRCA mutations or ectopic anatomy, the medical literature is deficient in a cohesive framework that juxtaposes their comprehensive genetic, histological, and etiological spectrum. This review fills the gap by compiling current information to evaluate how insights from breast cancer therapy might enhance vulvar cancer diagnostics and tailored therapies.

## 2. Materials and Methods

A comprehensive search of the PubMed, Scopus, and Oxford Journals Library databases was performed to locate peer-reviewed research published up to 2026. The search was performed between 25 July 2026 and 21 August 2026, according to the following terms: (a) ”Associations and similarities between breast cancer and vulvar cancer”; (b) “breast metastases from vulvar cancer”; and (c) “vulvar metastases from breast cancer”. Studies assessing epidemiological linkages, common genetic associations, or particular types of malignancies such as “Primary vulvar adenocarcinoma of mammary gland type (AMGT)” were included for qualitative synthesis. Related terms were also searched (e.g., “mammary-like adenocarcinoma of the vulva,” “AMGT,” or “vulvar metastasis”), and a total of around 550 articles were filtered according to the following criteria: English language, research done in humans, and research done in patients 18 years of age or above. Duplicates were removed, and the titles, abstracts and full texts were filtered and thereby included as per their relevance to the researched subjects.

Taking into account that the associations and similitudes between breast and vulvar benign and malignant lesions have not been commonly studied and that such research has not been extensively published in the medical literature, a very significant percentage of the findings of this review were from case studies, with this phenomenon constituting a limitation of the current review.

The search yielded results which were later grouped in seven main subjects, through the process of inductive thematic coding, as can be seen further in Section 3, the Results Section.

## 3. Results

Benign and malignant mammary-like vulvar tissues share hormonal receptors and biomarkers with orthotopic breast structures, but they differ from true metastatic pathways, HPV- or hormone-driven etiologies, lymphogenic recurrence routes, and site-specific prognostic systems.

The notions discussed in the following Section 3.1, Section 3.2, Section 3.3, Section 3.4, Section 3.5, Section 3.6 and Section 3.7 comprise the following key aspects:

- Benign lesions vs. malignant lesions. Breast-like tissue is a benign, ectopic vulvar remnant, whereas mammary-like adenocarcinoma is a rare primary in situ or invasive cancer of these specialized anogenital glands.
- Metastatic direction. Vulvar metastases from breast cancer (often lobular carcinoma via distant spread) contrast drastically with exceedingly uncommon breast metastases from vulvar cancer (which spreads by early locoregional lymphatics before rare hematogenous seeding).

Despite differing embryological and anatomical origins, both may express estrogen receptors (ERs) and progesterone receptors (PRs) and have overlapping luminal-like alterations, indicating endocrine responsiveness.

HPV participation in breast cancer is contentious, unproven, or minimal, although HPV is well established in common squamous vulvar tumors and seldom in specialized subtypes.

- Shared biomarkers. Both locations express ER, PR, GCDFP-15, and TRPS1, making immunohistochemistry necessary to distinguish initial vulvar sweat/mammary-type tumors from metastatic breast disease.
- Recurrence patterns. Breast cancer often has late systemic/visceral relapses (bone, liver, lungs), but vulvar tumors usually proceed locally and reappear in the inguinal–femoral lymph nodes.

Vulvar cancer prognosis depends on initial tumor size, depth of invasion, and inguinal lymph node status, whereas breast cancer prognosis depends on axillary node involvement, molecular subtype (luminal vs. triple-negative), and oncogenic scores.

### 3.1. Breast-like Tissue in the Vulva and Mammary-like Adenocarcinoma of the Vulva

Vulvar ectopic breast fibroadenoma (EBF) is a rare benign tumor with no clinical or radiological signs, making pre-operative distinction difficult, as presented by He [6]. The diagnosis is established by histopathology. Two non-exclusive histogenetic concepts are: (1) ectopic breast tissue along the embryonic “milk line,” and (2) hormonally sensitive anogenital mammary-like glands with latent potential for benign or malignant transformation. The co-existence of two benign tumors of the adenofibroma and adenoma type is rare and was documented by Robichaud [7].

Remaining mammary ridges that do not involute during embryologic development result in an uncommon disease known as ectopic breast tissue. Vulvar ectopic breast tissue can present with a wide range of clinical manifestations, depending on the amount and functional activity of the tissue involved. In the end, tissue biopsy and histopathologic analysis are used to make the diagnosis. There are no precise therapeutic recommendations since there is little research on supernumerary breast tissue on the vulva. Surgical removal is advised since this ectopic tissue has no use and might become a source of future cancer, as emphasized by Mikhael [8] in a statement based on limited case-level opinion rather than established guidance.

Several breast-like tumors are thought to be caused by supernumerary mammary glands generated from embryonic milk lines or vulvar mammary ridges. This phenomenon was reevaluated after recent findings of mammary-like anogenital glands (MLGs), a natural vulva component that seems to be closely connected to eccrine glands. A literature review by van der Putte [9] showed that “milk lines” originated in a mix of phylogenetic and ontogenetic theories at the start of this century and were not supported by observations in human embryos, which show that mammary gland primordia do not extend beyond the axillary–pectoral area. The “milk line” theory was also used more recently to explain the origin of tumors in studies by Babuccu [10] and Homsi [11]. The mammary ridges and milk lines cannot be used to determine vulvar MLGs since the breasts and vulva are so far apart. MLGs, which can also have eccrine or apocrine features, are the most likely source of lactating glands, lactating adenoma, fibroadenoma, hidrocystoma, hidradenoma papilliferum, and most cases of extramammary Paget’s disease and invasive adenocarcinoma in the anogenital region. Scurry [12] showed that MLG adenomas have diverse histological patterns and cell morphology. The most distinctive features are the ductal cell and a site compatible with or adjacent to the normal MLG. Variations of MLG adenomas include erosive, sclerosing, papillary, and syringocystadenoma papilliferans vulvar tumors.

When breast-like vulvar cancer is diagnosed, Fracchioli [13] recommends treating and following up similarly to in the case of orthotopic breast neoplasms, a conclusion shared also by Loo [1].

The characteristic “milk line” placement and its morphological and immunohistochemical similarities to breast cancer are needed to accurately diagnose AMGT, as underlined by Waugh [14]. Detecting tumor-associated benign mammary-like glands (DCIS) is crucial. The “mammary-like” character of AMGT and the exclusion of other vulvar primary and metastatic tumors require immunohistochemistry. The biologic nature of AMGT requires a specific therapeutic approach compared to other primary vulvar tumors.

Khan [15] emphasized that clinicians must be aware of accessory axillary breast tissue and its diseases because breast-screening exams do not include it. High-risk women may benefit from preventative excision.

Nobile [16] reported a rare case of simultaneous mammary-like vulvar and breast cancer. The paper highlights the diagnostic complexity of the case and the need for a personalized, interdisciplinary strategy based on tumor biology, staging, and patient comorbidities. The main aspects of breast-like tissue in the vulva and mammary-like adenocarcinoma of the vulva are summarized in Figure 1.

### 3.2. Metastases

#### 3.2.1. Vulvar Metastases from Breast Cancer

It is important to differentiate metastatic breast cancer from gynecologic original neoplasms and metastatic tumors from nearby GI and urinary systems, as shown by Braun [17].

Berghe’s [5] case was the first documented instance of vulvar metastases from breast cancer that was effectively treated with fulvestrant and abemaciclib, resulting in a full recovery. An important development in the treatment of vulvar metastases of breast origin is the use of CDK4/6 inhibitors, which provide a less intrusive and perhaps more successful alternative to existing treatment regimens.

The conclusion of a study by Khessairi [18] was that women with breast cancer must be closely monitored in order to find odd metastatic locations early and treat them appropriately.

In a case presented by Alligood Percoco [19], it was emphasized that the likelihood of delayed metastasis is heightened in individuals with invasive lobular carcinoma, especially among younger patients with hormone receptor-positive conditions. The current body of research indicates that these individuals are susceptible to postponed recurrence at remote metastatic locations.

#### 3.2.2. Breast Metastases from Vulvar Cancer

Metastatic breast cancer originating from initial vulvar carcinoma is very uncommon, with just a few recorded instances in the medical literature. From a clinical point of view, patients can themselves identify a lump in the breast that is either painless or uncomfortable, or they might find alterations in the breast tissue, or discharge from the nipple. Distinguishing breast metastasis from original breast cancer requires a comprehensive clinical history, histological assessment, and targeted immunohistochemistry staining to correlate the cells with the underlying vulvar tumor, an aspect emphasized by Vicus [20].

Breast and cutaneous metastases were described by Adhikari [21].

The majority of documented instances indicate that squamous cell (epidermoid) cancer of the vulva metastasizes to the breast tissue many months after the first therapy and that bilateral breast metastases from vulvar carcinoma are also possible.

Figure 2 depicts cross-links between primary cancer and metastases for the two sites discussed (breast and vulvar).

### 3.3. Genetic and Hormonal Connections

One tumor-suppressor gene that controls cell division is PTEN. Benign and malignant growths of several organs, including the breast, are predisposed to by pathogenic germline variations in PTEN. Dawson [22] highlighted that people with germline mutations in genes associated with cancer susceptibility are at an elevated risk of developing hyperplasia in any breast tissue, including rare ectopic sites.

Molecular testing revealed a pathogenic mutation of the AKT1 gene, a likely pathogenic frameshift insertion of the JAK1 gene, two likely pathogenic frameshift deletions of the KMT2C gene, and two variants of unknown significance (VUS) involving the ARID1A and OR2T4 genes, according to Lobrano’s [23] report on a mammary-like adenocarcinoma of the vulva. Ultimately, two BRCA1 gene CNVs were found. These findings were limited to the above-mentioned case report. Although this specific gene set is exclusive to Lobrano’s publication in the vulvar literature, elements such as the AKT1 mutation and KMT2C modifications are well-established oncogenic genes commonly found in orthotopic breast carcinomas, which MLAV resembles both morphologically and immunophenotypically, an aspect mentioned also by Grewal [24].

A specific BRCA2 mutation (8765delAG) was reported by Lamb [25] in a rare instance of mammary-like vulvar cancer occurring in a patient with a hereditary breast and ovarian cancer syndrome. Konstantinova [26] did not find any BRCA mutations in lesions involving anogenital mammary-like glands.

Synthetized aspects of molecular genetics in breast and vulvar tissue are presented in Figure 3.

### 3.4. HPV and Etiology

Both breast cancer and vulvar cancer have been associated with HPV to varying degrees with respect to the etiology of both diseases.

Cervical, vulvar, anal, and head and neck cancers are among the many malignancies that are known to be strongly linked to the human papillomavirus (HPV), as highlighted by Roman [27], the CDC Organization [28] and the NCI [29]. It is noteworthy that while HPV is linked to the aforementioned malignancies, its direct correlation with breast cancer is still up for discussion and investigation, as shown by Zhao [30], Kudela [31] and Anwer [32]. In a study on the same topic, Lin [33] came to the conclusion that national health records may be used to infer a relationship between HPV and breast cancer.

The prevalence of HPV in BC samples and its possible involvement in carcinogenesis were examined in Souza Wagner’s [34] research. It was estimated that overall, 23% of BC specimens had HPV. Oceania has the greatest regional incidence (38%) when broken down by continent. When BC tissues were compared to healthy controls, there was a noticeably higher chance of HPV discovery in the cancer group. South America had the strongest correlation among the continents. According to subgroup analysis based on economic categorization, the greatest incidence of HPV was found in low-income nations. Luminal B had the greatest HPV-positive rate according to evaluation by BC subtype. Karachalios’ [35] findings support the theory that HPV plays a role in the onset and progression of BC and suggest that HPV immunization may be useful in preventing BC.

As presented by Parra-Herran [36], the World Health Organization presently categorizes vulvar squamous cell carcinomas and their precursors according to their correlation with high-risk human papillomavirus (HPV). Driver changes in TP53 are often seen in HPV-independent lesions and are typically observed in the context of persistent vulvar inflammation. Nonetheless, a subset of pre-invasive vulvar squamous lesions is unrelated to both TP53 mutation and HPV. Significant acanthosis, verruciform growth, and altered squamous maturation are characteristics of the lesions described in this group, and over the last 20 years, a number of studies have contributed to their characterization. They have been linked to both conventional squamous cell carcinoma of the vulva and verrucous carcinoma, indicating a precursor function.

Li [37] claimed that individuals with HPV-associated vulvar cancer have a significantly different prognosis and are more sensitive to radiation than those with HPV-independent vulvar squamous cell carcinoma. Well-designed, adequately powered randomized controlled trials should be undertaken to assess the processes behind this enhanced sensitivity to radiation brought on by HPV. A group of patients suffering from HPV-independent disease and lacking p53 mutations had a better prognosis than p53-mutated forms, as shown by Fulgione [38].

The burden of HPV-associated vulvar illnesses may be significantly reduced by early detection, proper treatments, diligent follow-up, and preventative measures (such as vaccination), as highlighted by Swaminathan [39].

Although vaccination seems to lower recurrence rates after the first treatment of HPV-related lesions, as revealed by Montero-Macias [40], randomized studies have not established a definite adjuvant advantage. All things considered, HPV vaccine is quite successful in avoiding vulvar and vaginal diseases, particularly when given prior to HPV exposure.

Chehade [41] claims that considering HPV and p53 status while making decisions about locoregional therapy may help guide (de)escalation tactics. More research is needed to fully understand the function of immunotherapy, both alone and in combination, especially in the early stages of the illness. Inhibitors of the vascular endothelial growth factor, endothelial growth factor receptor, cell cycle, and DNA damage response are possible targeted therapies for advanced stages of VSCCs, especially in HPV-negative (HPV–) VSCCs (vulvar squamous cell carcinoma). In HPV-positive and HPV–/p53 wildtype VSCCs, it is considered a viable therapeutic strategy to target the phosphoinositide 3 kinase/mammalian target of rapamycin pathway, as presented by Chehade [41].

Oncogenic HPV types are essential for breast cancer and a major risk factor for vulvar cancer. HPV16 is the leading cause of tumors, followed by HPV18 (breast cancer) and HPV33 (vulvar cancer), as presented by Mirzoeva [42].

Several case–control studies have detected human papillomavirus (HPV) DNA in breast cancer tissues, indicating a potential biological link or indirect risk factor; nevertheless, comprehensive research has yet to establish a clear causative connection.

Figure 4 illustrates different aspects of HPV’s association with breast cancer and vulvar cancer.

### 3.5. Shared Biomarkers and Other Common Features

Both primary mammary-like adenocarcinomas of the vulva and breast carcinomas express the estrogen receptor (ER) and progesterone receptor (PR), as emphasized by Hu [43].

Muller [44] reported that HER2 positivity in VSCC is rare (2–5%) but insufficiently unexplored. Standardized evaluation utilizing modern ASCO/CAP breast, endometrial-specific, and/or gastric criteria is essential to determine HER2-positive vs. HER2-low/ultralow disease prevalence for HER2-targeted treatment.

Cytokeratin 7 (CK7), GATA3, and GCDFP-15 are used to detect vulvar tumors that originate from anogenital mammary-like glands as well as breast primary tumors, as shown by Lopez-Gonzalez [45].

For both forms of cancer, Ki-67 is a common comparative proliferation and prognostic marker.

Thankachan [46] found that long non-coding RNAs (lncRNAs) regulate biological processes, particularly in cancer formation. Breast and gynecologic malignancies (ovarian, cervical, endometrial, and vulvar) exhibit abnormal expression of nuclear paraspeckle assembly transcript 1 (NEAT1), which builds a framework for a nuclear body. By interacting with different regulatory proteins or sponging its corresponding tumor-suppressive microRNAs, highly expressed NEAT1 in breast and gynecologic malignancies often aids in the growth of tumors. NEAT1 is a promising therapeutic target in gynecologic and breast cancers due to its unique expression and role in tumorigenic pathways.

Prognostic significance: PDL-1 positivity is associated with more aggressive characteristics and worse overall survival in both breast cancer and vulvar squamous cell carcinoma (VSCC). Microenvironment: Expression is often seen in tumor-infiltrating immune cells (TILs/CPSs) rather than only in cancer cells. Subtype association (breast): This marker is significantly prevalent in triple-negative breast cancer (TNBC) and elevated histological grades. Subtype association (vulvar): It is observed in both HPV-related and HPV-unrelated VSCC, often associated with p53 modifications, as presented by Cirqueira [47], Rozenblit [48], Lotfinejad [49] and Baandrup [50].

Median tumor mutational burden (TMB) levels: Both cancer varieties often exhibit a low-to-moderate median TMB, averaging around 2.5 to 5 mutations per megabase, far lower than hypermutated tumors such as melanoma. Elevated TMB frequency: TMB-high classification (characterized by exceeding 10 mutations) (Mb) is present in a small fraction of patients—approximately 5% in breast carcinomas (an aspect studied by O’Meara [51] and Vougiouklakis [52]), with similarly limited groups in vulvar malignancies (Seino [53], Corey [54]). Immunotherapy efficacy: Due to the diminished overall mutational burden, tumor mutational burden (TMB) by itself is a less reliable predictor of single-agent checkpoint inhibitor efficacy in these populations than the PDL-1 combined positive score (CPS) or the density of active tumor-infiltrating lymphocytes (TILs), facts supported by Barroso Sousa [55] and Choschzick [56].

Breast cancer with elevated tumor mutational burden (TMB) subgroups, especially in triple-negative breast cancer (TNBC), strongly support the use of immune checkpoint inhibitors owing to enhanced neoantigen display. Vulvar squamous cell carcinoma: The efficacy of immunotherapy is contingent upon intricate microenvironment characteristics and PDL-1 expression profiles, rather than only on TMB, as the clinical pathways of vSCC often advance irrespective of rigid TMB criteria.

Figure 5 details the molecular and immunological features shared by primary mammary-like adenocarcinomas of the vulva and breast carcinomas.

### 3.6. Recurrence Patterns

Both breast cancer and vulvar cancer demonstrate common recurrence trends characterized by an initial heightened risk of local and regional lymph node relapse, a considerable dependence on primary nodal status as a prognostic factor, and an infrequent yet significant potential for late or atypical metastatic events.

#### 3.6.1. Local and Regional Dominance

Main relapse area: Both tumors often recur at or adjacent to the initial neoplasm location or local lymph node regions before spreading throughout the body, as shown by Zach [57].

Lymphatic dissemination: The engagement of regional lymph nodes is the foremost predictor of recurrence in both forms of the illness, as presented by Woelber [58].

Temporal clustering: A significant percentage of locoregional recurrences for both breast and vulvar cancers occur during the first two to three years after therapy, as shown by Rohrmoser [59].

#### 3.6.2. Metastatic and Biological Parallels

Remote dissemination: When remote failure occurs, it often manifests as a consequence of or concurrently with unchecked localized illness; however, certain variants (such as lobular breast carcinoma) may exhibit atypical metastatic preferences, as underlined by Yu [60].

Anatomical intersections: Notably, initial breast carcinoma may unusually metastasize to the vulva, whereas few primary mammary-like neoplasms of the vulva emulate the biology and immunohistochemical recurrence characteristics of breast cancer, as explained by Nobile [16].

Figure 6 summarizes shared and unique recurrence patterns in breast cancer and vulvar cancer.

### 3.7. Prognostic Factors

The most common type of vulvar cancer is squamous cell carcinoma, whereas breast cancer is most often an adenocarcinoma, but they share many clinical and prognostic features, especially regarding the importance of regional lymph node status in determining survival, and biological similarities in rare mammary-type tumors of the vulva that express hormone receptors and GATA3.

#### 3.7.1. Prognostic Factors and Survival Similarities

Lymph node involvement: In the cases of vulvar and breast cancer, the condition of regional lymph nodes (inguinofemoral for vulvar cancer, axillary for breast cancer) serves as the most significant and reliable independent prognostic indicator for overall and disease-free survival, as explained by Khullar [61].

Tumor staging and burden: Improved anatomical categorization, larger initial tumor dimensions and higher invasion depth or stromal infiltration are substantially related to/associated with increased recurrence probability and worse 5-year survival rates in both cancers, as emphasized by Miljanovic-Spika [62].

Histological grading: An elevated tumor grade (poor differentiation) serves as a negative prognostic marker, indicating more aggressive biological characteristics and an increased probability of local or distant recurrence, an opinion shared by Liu [63].

#### 3.7.2. Clinical Associations and Metastatic Overlap

Metastatic mimicry and locations (see Figure 7): Breast cancer may seldom migrate to the vulva, often years after initial therapy, indicating extensive systemic illness. In contrast, vulvar malignancies may seldom spread to breast tissue, as shown by Khessairi [18].

Mammary-like adenocarcinoma of the vulva (MLAV) is a rare subset of vulvar tumors that develops from anogenital mammary-like glands. These tumors display morphological and immunohistochemical similarities to primary breast cancer, such as positive estrogen/progesterone receptors and GATA3 expression. Due to these similarities, clinicians often utilize prognostic profiling and targeted treatment algorithms modeled after breast cancer, as discussed by Tessier-Cloutier [64].

Biomarker correlations: Modifications in pathways such as TP53 or PIK3CA affect prognosis and recurrence probabilities in both tumor categories, underscoring common oncogenic factors in specific clinical groupings.

## 4. Discussion

This study focuses on an uncommon, but clinically significant, junction of breast and vulvar disease. This association includes a number of different phenomena: spontaneous mammary-like tumors of the vulva, malignant transformation in ectopic breast tissue, metastasis between the breast and vulva, and partly similar molecular or immunological aspects. Despite their comparable appearance and biomarker expression patterns, these entities vary significantly as far as histogenesis, clinical behavior, and treatment implications are concerned. Thus, precise categorization is crucial to prevent misdiagnosis and incorrect therapy. A major controversy relates to the development of breast-like tissue and mammary-type tumors of the vulva. These lesions have been classically described as remains of the embryonic milk line. However, this idea has been contested, since no solid evidence has been found for the extension of human mammary ridges into the anogenital area. The alternative notion that these tumors emerge from naturally existing anogenital mammary-like glands is gaining growing acceptance. These glands show morphological and functional similarities to mammary, apocrine and eccrine structures and may develop benign tumors such as fibroadenoma, lactating adenoma and hidradenoma papilliferum and invasive adenocarcinoma and extramammary Paget disease. However, these two ideas may not be mutually exclusive in every case, and the exact origin of a single lesion cannot always be determined by routine histology alone. The most difficult differential diagnosis is between primary mammary-like adenocarcinoma of the vulva and metastatic breast cancer, as illustrated in Table 1. Both may be positive for glandular morphology and for ER, PR, GATA3, CK7 and GCDFP-15. These indicators facilitate mammary differentiation, although none is absolutely specific for a primary tumor in the breast. Therefore, diagnosis should include the clinical history of the patient, mammography, tumor location, microscopic findings, immunohistochemical profile and evidence of adjacent benign mammary-like glands or in situ components. The presence of ductal carcinoma in situ-like alterations or continuity with normal anogenital mammary-like glands favors primary vulvar origin. However, the existence of a breast cancer history, systemic spread, and close morphological or molecular concordance between breast and vulvar tumors favors metastatic illness. Whenever there is ambiguity, an analysis of comparative molecular profiling of both cancers may assist in determining whether they are of similar clonal origin.

The reviewed data also suggests that breast cancer may metastasize to the vulva, although this is quite rare. Vulvar metastasis may present late in the course of breast cancer and may mimic a primary vulvar tumor or a benign lesion. This hypothesis is particularly pertinent in individuals with invasive lobular carcinoma, a subtype known to have atypical metastatic patterns. Therefore, a new vulvar lump in a woman with a history of breast cancer should be biopsied rather than believed to be a primary gynecological illness. Similarly, histological verification is required for breast lesions developing in individuals previously treated for vulvar cancer, since rare breast metastases from vulvar carcinoma have been documented. Because these lesions are metastatic, the implications for treatment are essential, as therapy should be directed primarily at the systemic sickness rather than at local excision alone. The observed response of vulvar metastases of breast origin to fulvestrant and abemaciclib raises the possibility of a potential benefit of biomarker-directed systemic treatment. In general, the management of mammary-like vulvar adenocarcinoma has often been extrapolated from breast cancer procedures due to its rarity and biological similarities to mammary carcinoma. Localized illness is still mostly treated surgically, with consideration given to sentinel lymph node biopsy, radiation, endocrine therapy, chemotherapy or HER2-targeted treatment depending on stage and tumor profile. Direct use of breast cancer algorithms, however, must be done with caution. The vulva has distinct anatomical drainage, surgical restrictions and patterns of local recurrence. Moreover, the data supporting breast-based therapy is mostly from individual cases and small case series. Therefore, a multidisciplinary evaluation that includes gynecologic oncologists, breast specialists, pathologists, radiologists, geneticists, and medical oncologists is recommended. Shared biomarkers may provide more treatment prospects, but their interpretation should be done in the right clinical and pathological context. Testing of ER and PR expression may support the use of endocrine therapy in mammary-like vulvar adenocarcinoma, whereas HER2 testing may identify a small minority of patients who may benefit from HER2-directed therapy. Standardized testing is necessary since discrepancies across breast, gastric, and gynecologic scoring methods might modify the categorization of HER2-positive, HER2-low, or HER2-ultralow illness. Similarly, PD-L1 expression and tumor-infiltrating lymphocytes may be used to identify patients that may benefit from immunotherapy. In breast and vulvar cancers, the tumor mutational load is often low to moderate and does not seem adequate as a single prognostic biomarker. A composite score including PD-L1, MSI, genomic changes, immune-cell infiltration and clinical features is likely to be more useful than any single marker. Molecular discoveries reviewed in this research suggest that there may be similar oncogenic pathways in specific breast and vulvar cancers, rather than a common genetic basis. Alterations in PTEN, AKT1, JAK1, KMT2C, BRCA1, TP53 and PIK3CA signaling may play a role in tumor initiation or progression. These studies justify extensive molecular testing in atypical, recurring, or advanced mammary-like vulvar tumors, especially when results may alter targeted therapy or suggest genetic cancer risk. Germline testing may also be indicated for individuals with multiple primary malignancies, significant family history, early-onset illness or tumors originating in ectopic mammary-type tissue. However, the genomic data available are from very few cases, and a characteristic molecular hallmark of mammary-like adenocarcinoma of the vulva has not yet been characterized. Special caution is required in the understanding of the etiological function of HPV. The causal association of high-risk HPV with a significant number of vulvar squamous cell carcinomas is well established, although the involvement of HPV in breast carcinogenesis is debatable. The reports of HPV DNA in breast tumors and the relationships in the meta-analyses are not in themselves evidence of causation. The great variation across research might be attributed to differences in specimen preservation, geographic dispersion, laboratory procedures, contamination control, and the choice of comparator tissues. Thus, HPV-associated and HPV-independent routes are clinically relevant in vulvar squamous cell carcinoma, but a similar etiological categorization cannot be extended to breast cancer at present. The established preventive effect of HPV vaccination on HPV-related anogenital illness should continue to be promoted, and any decrease in breast cancer risk should be considered hypothetical until more prospective and mechanistic data is obtained. Breast and vulvar tumors are biologically distinct; however, there are numerous similar concepts of prognosis. Regional lymph node involvement is a primary driver of recurrence and survival in both illnesses, while tumor size, stage, depth of invasion, histological grade and biological subtype further clarify risk. Both cancers are characterized by a prevalence of early locoregional recurrence, although late relapse and unusual metastatic locations might occur. These parallels justify thorough monitoring, adjusted for baseline nodal status, tumor biology, treatment history, and symptoms. However, follow-up measures should be disease-specific, since absolute risks, anatomical patterns and available data vary greatly between conventional breast cancer, vulvar squamous cell carcinoma and mammary-like vulvar adenocarcinoma. Limitations of this review include the rarity and variability of the studied diseases. Most available data consists of case reports, tiny retrospective series, and narrative reviews, with a high risk of publishing and selection bias. Negative or clinically unremarkable instances are less likely to be reported than successful therapies and atypical metastatic patterns. Terminology has also changed over time, making comparability between research problematic, especially for ectopic breast tissue, mammary-like anogenital glands and mammary-type vulvar cancer.

Future research should concentrate on worldwide registries and multicenter cooperation to get standardized clinical, histological, immunohistochemical, molecular, therapeutic and survival data. Comparative genomic studies of primary breast tumors, primary mammary-like vulvar adenocarcinomas, and vulvar metastases may provide insight into their histogenesis and discover diagnostically important genetic markers. Consensus criteria are also required to differentiate between primary vulvar mammary-like tumors and metastatic breast cancer and other vulvar glandular malignancies. Due to the modest numbers, prospective randomized trials are unlikely to be performed, but properly structured registries and molecularly documented case series might give more robust data than isolated case reports.

The present documented and updated study of various and heterogeneous data does not provide proof of a unifying biological link between breast and vulvar cancer; rather, this review offers a qualitative narrative synthesis of evidence.

## 5. Conclusions

To summarize, the conclusion must be calibrated to a level of caution triggered by the fact that case reports represented an important part of the bibliography, given the scarcity of the subject studied in this present literature review. Nevertheless, the connection between breast and vulvar cancer is rare but clinically significant. Recognition relies on differentiation of actual metastatic disease from original tumors emerging in anogenital mammary-like glands and other vulvar neoplasms with overlapping morphologic features. Histopathological investigation is still crucial, but a successful diagnosis sometimes involves clinical correlation, targeted immunohistochemistry and imaging and, where applicable, genetic testing. Therapeutic choices should be based on the type, stage, receptor profile and genetic features of the tumor and the general state of the patient, rather than on anatomical location alone. Personalized multidisciplinary care is crucial.

## Figures and Tables

**Figure 1:**
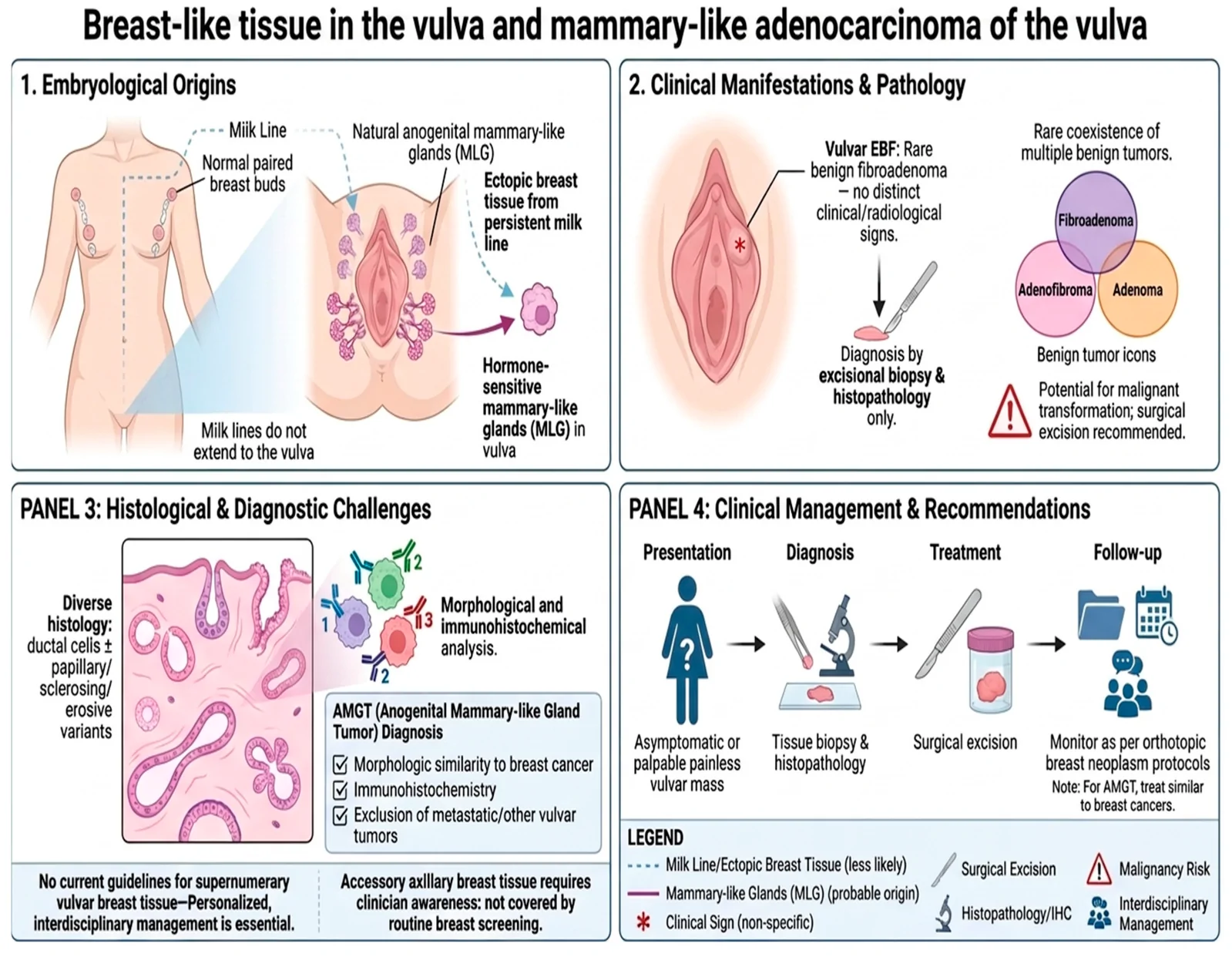
Breast-like tissue in the vulva and mammary-like adenocarcinoma of the vulva.

**Figure 2:**
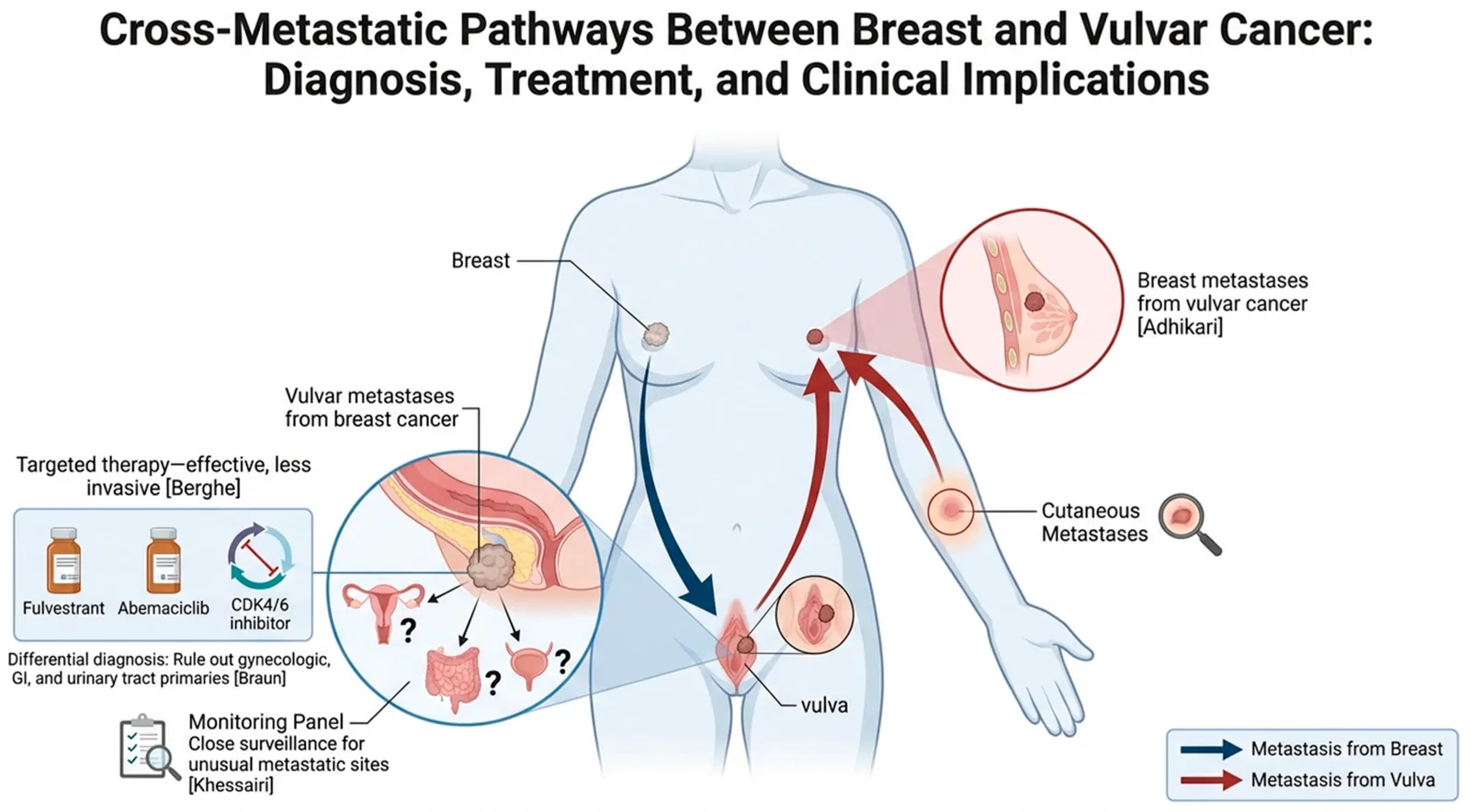
Cross-metastatic pathways between breast and vulvar cancer: diagnosis, treatment and clinical implications. The question mark symbolizes the dilemma in the differential diagnosis of a secondary tumor situated at the level of the vulva.

**Figure 3:**
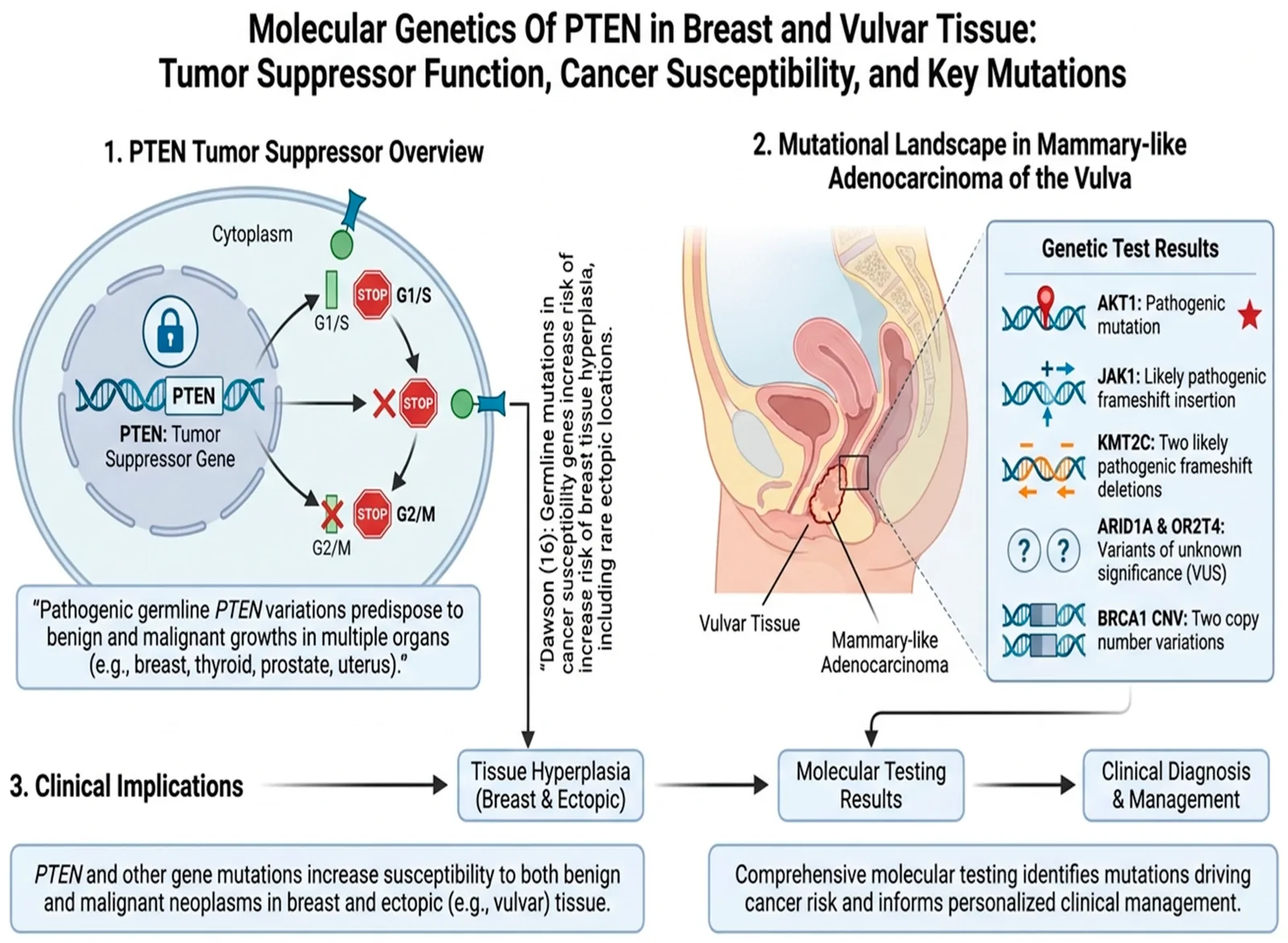
Breast and vulvar tissue—molecular genetics and cancer susceptibility. In the fields of human genetics and oncology, AKT1, JAK1, KMT2C, and BRCA1 represent unique physiological pathways that may experience neoplastic disruption in breast and vulvar tissues. Their roles are often evaluated in comparison to PTEN, a principal regulatory tumor suppressor.

**Figure 4:**
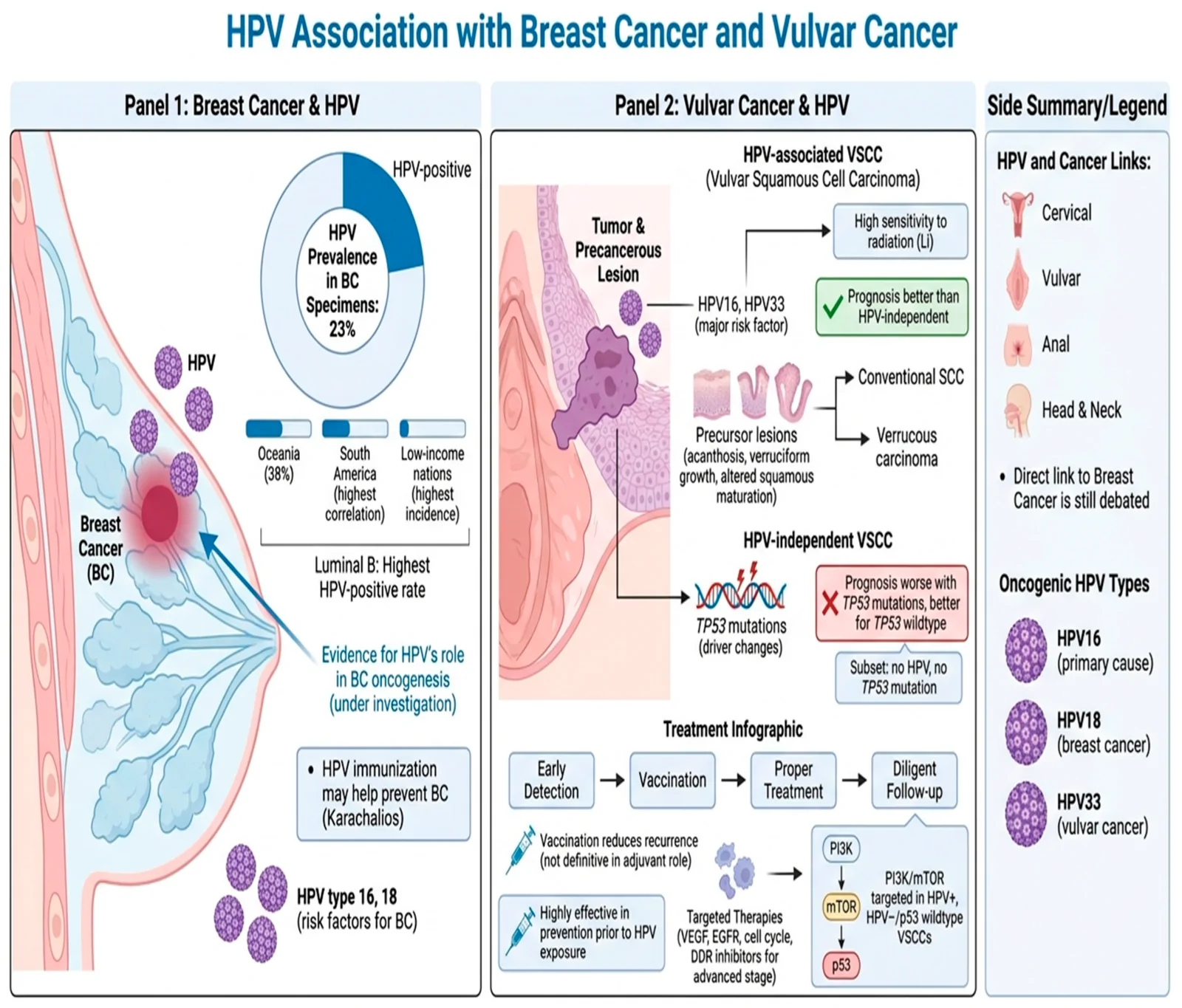
HPV’s associations with breast cancer and vulvar cancer: links, treatments and debated connections.

**Figure 5:**
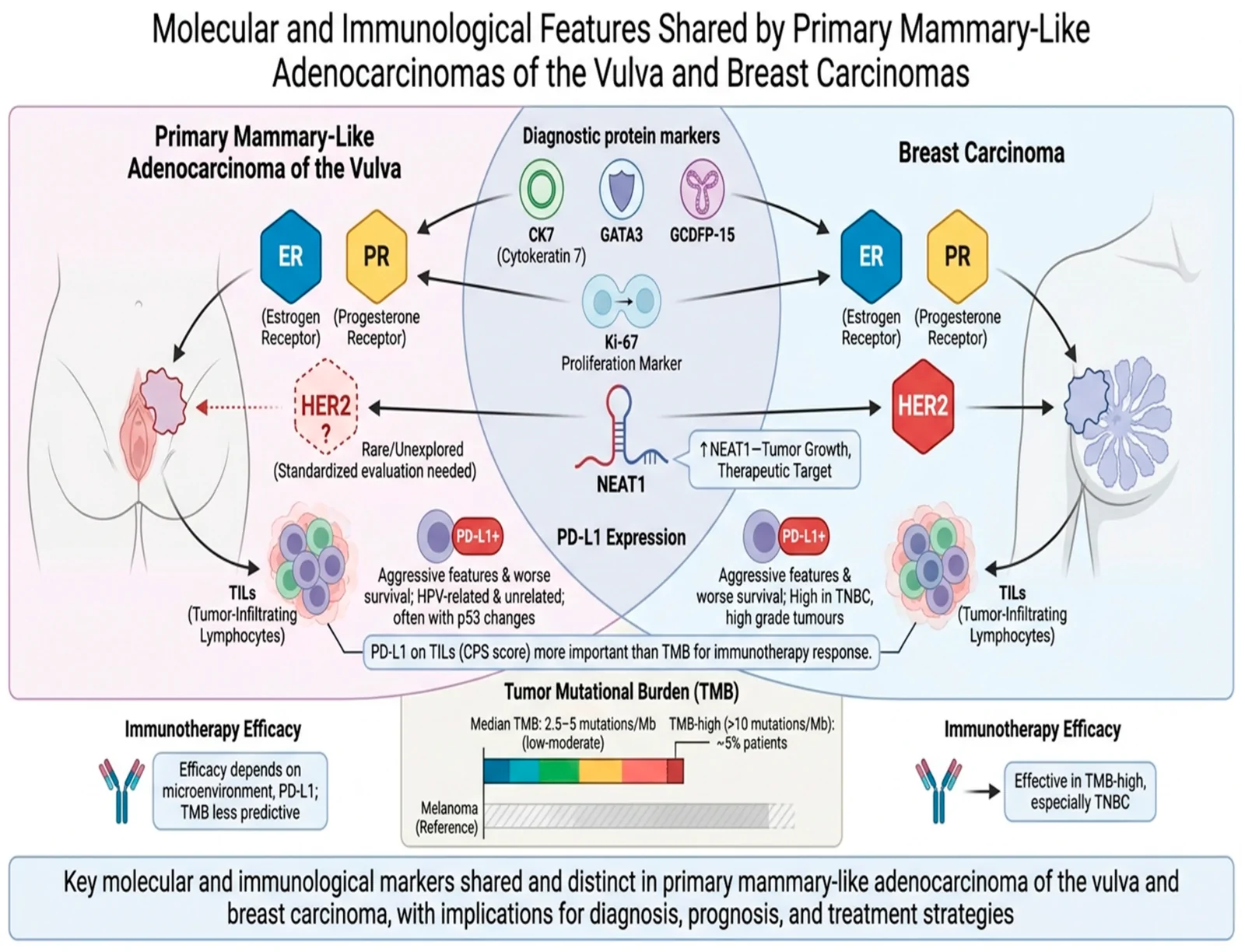
Molecular and immunological features shared between primary mammary-like adenocarcinoma of the vulva and breast carcinoma. The term “rare” refers to a pooled HER2 overexpression of 2–5% in vulvar squamous cell carcinoma.

**Figure 6:**
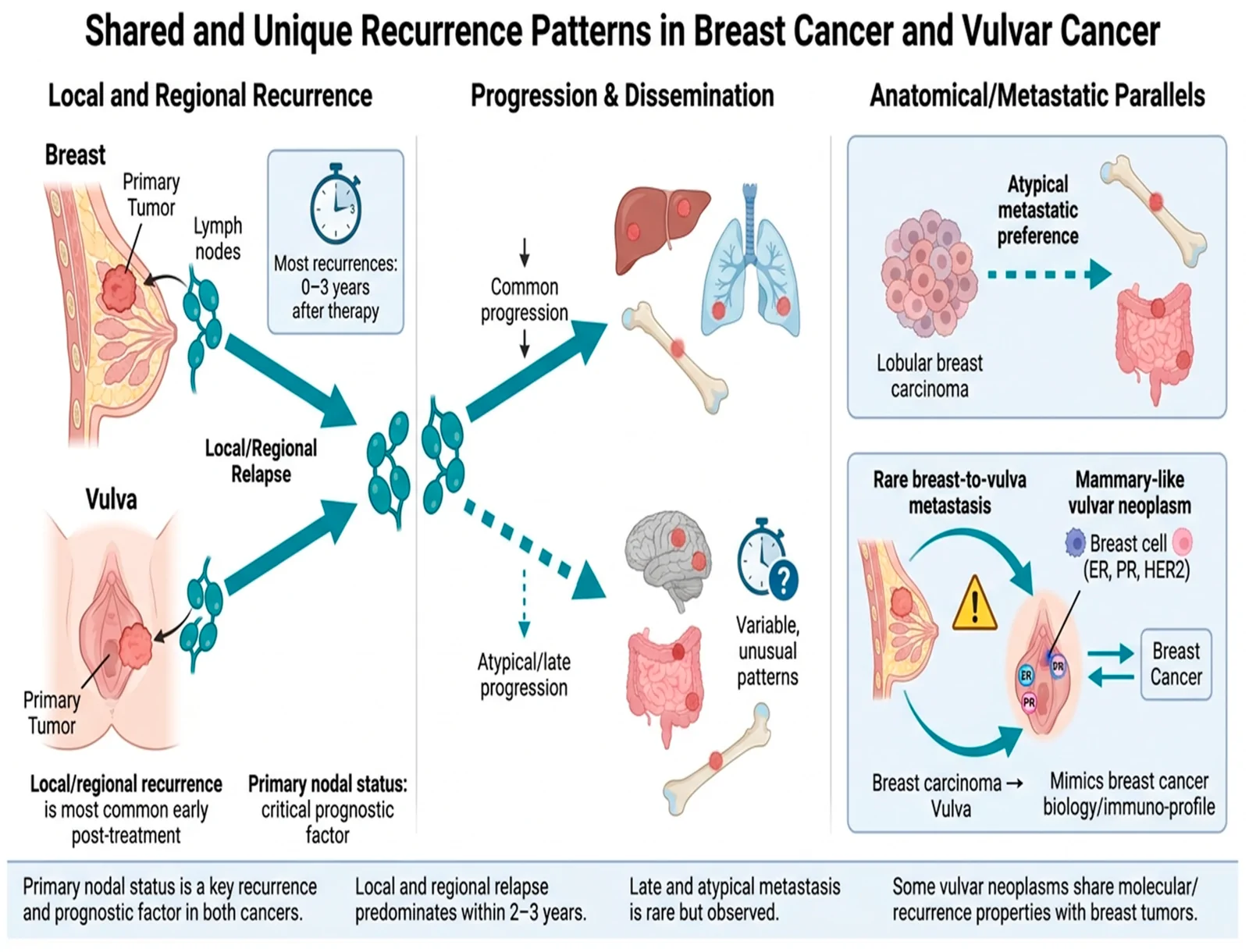
Shared and unique recurrence patterns in breast cancer and vulvar cancer.

**Figure 7:**
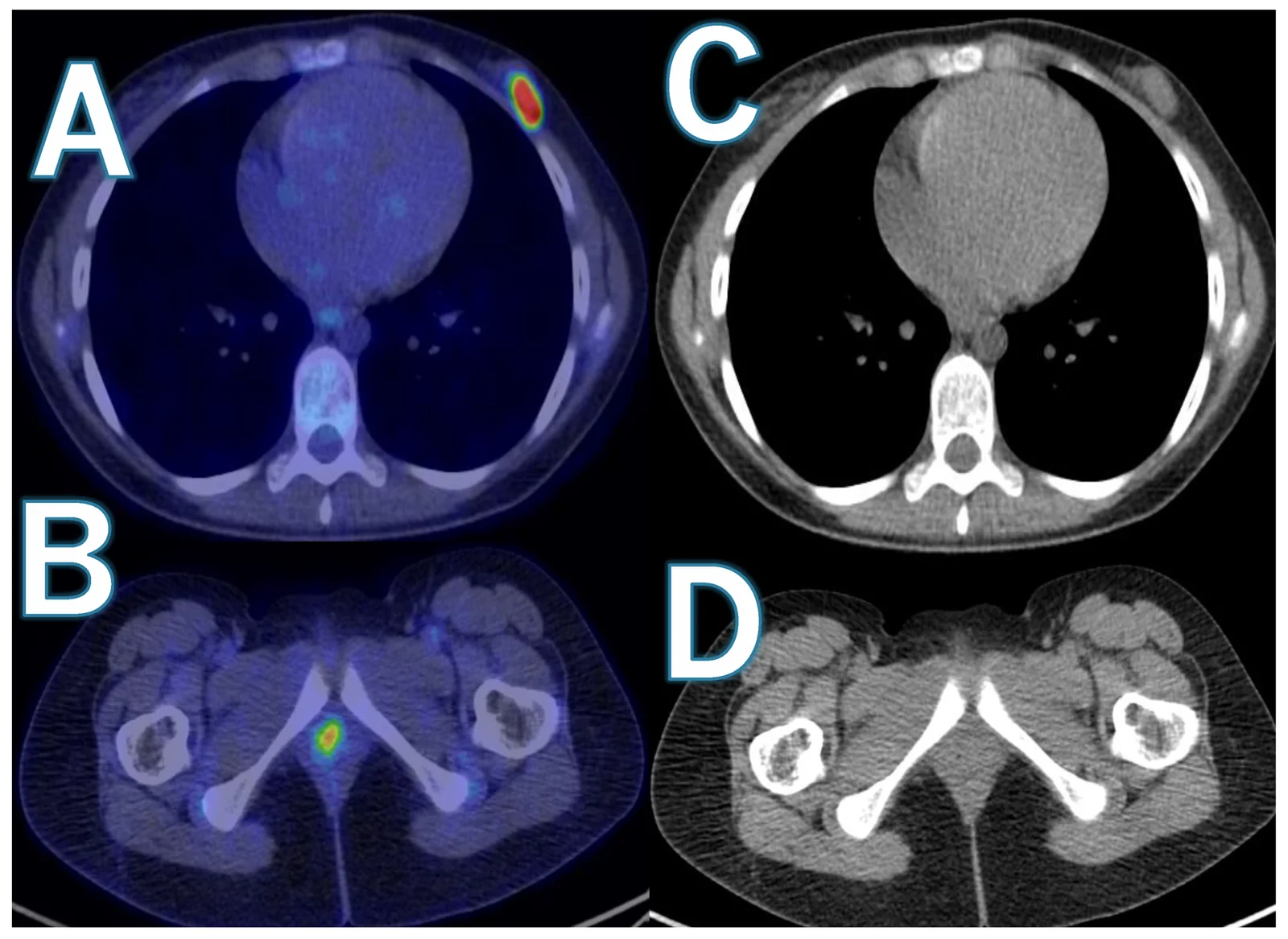
Metastatic vulvar carcinoma at the level of the breast. A 63-year-old woman with a right labia minora mass and right inguinal lymphadenopathy underwent surgery in December 2022 for FIGO stage IIIA vulvar cancer, followed by adjuvant radiotherapy. Two years after completion of treatment, a local recurrence was identified, along with a metabolically active mass in the lower outer quadrant of the left breast on PET-CT. An ultrasound-guided core needle biopsy of the breast lesion confirmed metastatic vulvar carcinoma. Figure 7 orientation parameters: Firstly, the two images in the superior row (**A**,**C**) illustrate transverse sections at the level of the thorax, while the images in the inferior row (**B**,**D**) illustrate transverse sections at the level of the perineum; secondly, the two images (**C**,**D**) on the viewer’s right represent CT images, while the two images on the viewer’s left (**A**,**B**) represent PET images. This figure originates from the authors’ clinical cases, and the patient signed an informed consent form regarding the use of these images for science and/or research.

**Table 1:** Comparative aspects between primary mammary-like adenocarcinoma of the vulva and metastatic breast cancer.

Comparison: Primary Mammary-like Adenocarcinoma of the Vulva vs. Metastatic Breast Cancer		
Feature	Primary Mammary-Like Adenocarcinoma of Vulva (AMGT)	Metastatic Breast Cancer to the Vulva
Origin	Anogenital mammary-like glands or primitive mammary ridge remnants in the vulva (commonly labia majora).	Orthotopic breast tissue (primary breast carcinoma).
Clinical History	No prior or concurrent history of breast cancer; thorough breast imaging/exam shows no primary breast lesion.	History or concurrent diagnosis of breast cancer (often widely metastatic at presentation).
Adjacent Histology	Presence of benign/residual normal mammary-like glands or in situ changes adjacent to the tumor.	Absence of benign/residual mammary-like glands in the vulvar stroma; pure metastatic deposits.
Histopathology	Morphologically identical to breast carcinoma (ductal, lobular, mucinous, etc.).	Matches the histology of the primary breast tumor (e.g., invasive lobular carcinoma often metastasizes to gynecologic sites).
Immunohistochemistry	Positive for GATA-3, ER, PR, and commonly TRPS1; negative for markers like CK20, CDX2, and PAX8.	Positive for GATA-3, ER, and PR (usually mirroring the primary breast profile); clinically interpreted alongside breast markers.
Initial Staging/Spread	Frequently presents as a local vulvar mass with potential inguinofemoral lymph node involvement; distant metastasis is rare at presentation.	Often part of multi-organ or widespread systemic dissemination (bone, viscera, etc.).
Management Focus	Wide local excision or radical vulvectomy with regional lymph node assessment; systemic/hormonal therapy if hormone receptor-positive.	Systemic chemotherapy, targeted therapy, and/or hormone therapy prioritized; local vulvar surgery reserved for palliation or select cases.

## Data Availability

The original contributions presented in this study are included in the article. Further inquiries can be directed to the corresponding authors.

## References

[1] Loo K.H., Hwang M.J. (2026). Ectopic breast cancer in vulva and other non-axillary sites: A systematic review. World J. Surg. Oncol..

[2] Tan N., Wu Y., Li B., Chen W. (2024). Burden of female breast and five gynecological cancers in China and worldwide. Chin. Med. J..

[3] Papatheodorou D.C., Liakou C.G., Kalogerakos K., Dimopoulos J.C.A., Kalinoglou N. (2017). Bilateral Breast Metastases from Vulvar Carcinoma: A Case Report and Literature Review. Case Rep. Obstet. Gynecol..

[4] Zhang Z., Li Y., Huang H., Wei T., Huang Y., Qu X., Xu Y., Zhang A., Li J., Gong Z. (2025). Disparities and trends of the incidence and mortality of female-specific cancers in the United States. PLoS ONE.

[5] Berghe C.V., Antxustegi A.L., Nicaise N., Borght T.V., D’HOndt L., Moerman J., de Codt M. (2026). Vulvar Metastasis from Breast Cancer Treated with Abemaciclib plus Fulvestrant: A Case Report. Case Rep. Oncol..

[6] He Y., Xie G., Ji Y., Shi Y., He Y., Lei X. (2025). Ectopic breast fibroadenoma of the vulva: A case report. Front. Oncol..

[7] Robichaud S., Tran-Thanh D., Zhang Z.P., Durocher F., Rahimi K. (2025). Lactating Vulvar Adenoma Associated with Fibroadenoma. Int. J. Surg. Pathol..

[8] Mikhael S., Nilsson W., Patel K., Graf S. (2017). Ectopic Breast Tissue of the Vulva in a Postmenopausal Woman. Case Rep. Obstet. Gynecol..

[9] Van Der Putte S.C.J. (1994). Mammary-like glands of the vulva and their disorders. Int. J. Gynecol. Pathol..

[10] Babuccu O., Kalayci M., Turhan E., Gun B.D., Dursun A. (2012). Mammae erraticae: A case report and reappraisal of the related theories. Aesthetic Plast. Surg..

[11] Homsi H.A., Sharma A., Przybycin C., Piliang M. (2024). Fibroadenoma of the vulva with pseudoangiomatous stromal hyperplasia: A common neoplasm in uncommon site. J. Cutan. Pathol..

[12] Scurry J., Van Der Putte S.C.J., Pyman J., Chetty N., Szabo R. (2009). Mammary-like gland adenoma of the vulva: Review of 46 cases. Pathology.

[13] Fracchioli S., Puopolo M., DE LA Longrais I.R., Scozzafava M., Bogliatto F., Arisio R., Micheletti L., Katsaros D. (2006). Primary ‘breast-like’ cancer of the vulva: A case report and critical review of the literature. Int. J. Gynecol. Cancer.

[14] Waugh S.B., Maku H.O., Antosh D.D., Rozycki S.K., Kamat A.A., Okoye E.I., Coffey D.M., Deavers M.T., Schwartz M.R., Ge Y. (2026). A rare case of multifocal vulvar carcinoma of mammary gland type (AMGT) with mucinous features: Differential diagnosis and literature review. Gynecol. Oncol. Rep..

[15] Khan R.N., Parvaiz M.A., Khan A.I., Loya A. (2019). Invasive carcinoma in accessory axillary breast tissue: A case report. Int. J. Surg. Case Rep..

[16] Nobile I., Tessitore A., Palumbo M., Boccia D., Mignogna C., Bifulco G., Della Corte L. (2026). A case report of a mammary gland type adenocarcinoma of the vulva in a patient with a concomitant breast cancer: A diagnostic challenge. Front. Oncol..

[17] Braun A., Reddy S., Cheng L., Gattuso P., Yan L. (2023). Clinicopathologic Review of Metastatic Breast Cancer to the Gynecologic Tract. Int. J. Gynecol. Pathol..

[18] Khessairi N., Sakhri S., Chrigui M., Krimi O., Bouaziz H., Dhiab T.B. (2025). Breast Metastasis from Vulvar Carcinoma: Case Report and Review of Literature. Case Rep. Obstet. Gynecol..

[19] Alligood-Percoco N.R., Kessler M.S., Willis G. (2015). Breast cancer metastasis to the vulva 20 years remote from initial diagnosis: A case report and literature review. Gynecol. Oncol. Rep..

[20] Vicus D., Korach J., Friedman E., Rizel S., Ben-Baruch G. (2006). Vulvar cancer metastatic to the breast. Gynecol. Oncol..

[21] Adhikari N., Vennepureddy A., Parylo S., Agarwal A., Dhar M. (2021). Vulvar Cancer with Cutaneous and Breast Metastases. Case Rep. Med..

[22] Dawson H., Smrke A., Ellery P.M., Wilkinson N., Rosenthal A.N., McVeigh T.P. (2022). Fibroadenoma in vulval ectopic breast tissue in a patient with PTEN Hamartoma Tumour Syndrome. Fam. Cancer.

[23] Lobrano R., Manca A., Sini M.C., Palmieri G., Petrillo M., Cossu A., Paliogiannis P. (2023). Mammary-like adenocarcinoma of the vulva: A rare case report with next generation sequencing. Pathologica.

[24] Grewal J.K., Eirew P., Jones M., Chiu K., Tessier-Cloutier B., Karnezis A.N., Karsan A., Mungall A., Zhou C., Yip S. (2017). Detection and genomic characterization of a mammary-like adenocarcinoma. Cold Spring Harb. Mol. Case Stud..

[25] Lamb A., Darus C.J., Skripenova S., Weisberg T., Miesfeldt S. (2013). Association of primary breast cancer of the vulva with hereditary breast and ovarian cancer. J. Clin. Oncol..

[26] Konstantinova A.M., Shelekhova K.V., Imyanitov E.N., Iyevleva A., Kacerovska D., Michal M., Kazakov D.V. (2017). Study of Selected BRCA1, BRCA2, and PIK3CA Mutations in Benign and Malignant Lesions of Anogenital Mammary-Like Glands. Am. J. Dermatopathol..

[27] Roman B.R., Aragones A. (2021). Epidemiology and incidence of HPV-related cancers of the head and neck. J. Surg. Oncol..

[28] Basic Information About HPV and Cancer|Cancer|CDC. https://www.cdc.gov/cancer/hpv/basic-information.html.

[29] HPV and Cancer—NCI. https://www.cancer.gov/about-cancer/causes-prevention/risk/infectious-agents/hpv-and-cancer.

[30] Zhao G., Chang J., Wei K. (2024). Correlation between breast cancer and human papillomavirus (HPV) infection. Heliyon.

[31] Kudela E., Kudelova E., Kozubík E., Rokos T., Pribulova T., Holubekova V., Biringer K. (2022). HPV-Associated Breast Cancer: Myth or Fact?. Pathogens.

[32] Anwer M., Bhaliya K., Zahra M., Virk U.Y., Malik H.A., Wei M.Q. (2026). Therapeutic Landscape of HPV-Associated Cancers: From Mechanisms and Conventional Approaches to Future Innovations. Cancers.

[33] Lin C., Tsai S.C.S., Huang J.Y., Lin F.C.F. (2023). HPV infection and breast cancer risk: Insights from a nationwide population study in Taiwan. Front. Oncol..

[34] de Souza Wagner P.H., Matheus G.T.F.U., Menegat B.L.R.S., Menegat A.L.R.S., Ribeiro D.M., de Moraes F.C.A. (2026). HPV in breast cancer: Prevalence and comparison with healthy tissue-a systematic review and meta-analysis. Cancer Causes Control.

[35] Karachalios C., Petousis S., Margioula-Siarkou C., Dinas K. (2023). Human papillomaviruses and breast cancer: A systematic review and meta-analysis. Oncol. Lett..

[36] Parra-Herran C., Nucci M.R., Singh N., Rakislova N., Howitt B.E., Hoang L., Gilks C.B., Bosse T., Watkins J.C. (2022). HPV-independent, p53-wild-type vulvar intraepithelial neoplasia: A review of nomenclature and the journey to characterize verruciform and acanthotic precursor lesions of the vulva. Mod. Pathol..

[37] Li W., Zhai L., Zhu Y., Lou F., Liu S., Li K., Chen L., Wang H. (2024). Review effects of radiation treatment on HPV-related vulvar cancer: A meta-analysis and systematic review. Front. Oncol..

[38] Fulgione C., Inzani F., Raffone A., Raimondo D., Arciuolo D., Ronchi S., Marchiori D., Maragliano R., Neola D., Vastarella M.G. (2025). Prognosis of HPV-independent, p53-wild-type vulvar squamous cell carcinoma: A systematic review and meta-analysis. Gynecol. Oncol..

[39] Swaminathan S., Forman J., Mercurio M.G. (2026). HPV-related Vulvar Disease. Clin. Obstet. Gynecol..

[40] Montero-Macías R., Adam M., Rouzier R., Bonneau C. (2025). HPV vaccination efficacy in primary and tertiary prevention of vulvar and vaginal HPV-related high grade dysplasia and cancers: A systematic review. Hum. Vaccin. Immunother..

[41] Chehade R., Jerzak K.J., Tavanger F., Plotkin A., Gien L.T., Leung E., Mackay H. (2025). Advances in Vulvar Cancer Biology and Management. J. Clin. Oncol..

[42] Mirzoeva J.V., Shakova A.R., Karaeva E.M., Slugina V.A., Abdullayeva S.A. (2025). Type-Specific Association of Highly Oncogenic Human Papillomavirus Genotypes with the Risk of Squamous Cell Carcinoma of the Skin of the External Female Genitalia and Invasive Cervical Cancer: Meta-Analysis. Reprod. Health East. Eur..

[43] Hu L., Tiesinga J. (2024). Case report: Primary vulvar adenocarcinoma of mammary gland type—Its genetic characteristics by focused next-generation sequencing. Pathol. Oncol. Res..

[44] Müller N.L.F., Al Sibani M., Ayoub Y., Ayoub M., Pullattayil A.K., Tavangar F., Plotkin A., George S., Jerzak K.J., Mackay H. (2026). HER2 Expression in Squamous Cell Carcinoma of the Vulva: A Systematic Review and Meta-Analysis. Cancers.

[45] Lopez-Gonzalez L., Cendra A.S., Cendra C.S., Cervantes E.D.R., Espinosa J.C., Pekarek T., Fraile-Martinez O., García-Montero C., Rodriguez-Slocker A.M., Jiménez-Álvarez L. (2024). Exploring Biomarkers in Breast Cancer: Hallmarks of Diagnosis, Treatment, and Follow-Up in Clinical Practice. Medicina.

[46] Thankachan S., Bhardwaj B.K., Venkatesh T., Suresh P.S. (2021). Long Non-coding RNA NEAT1 as an Emerging Biomarker in Breast and Gynecologic Cancers: A Systematic Overview. Reprod. Sci..

[47] Cirqueira M.B., Mendonça C.R., Noll M., Soares L.R., Cysneiros M.A.d.P.C., Paulinelli R.R., Moreira M.A.R., Freitas-Junior R. (2021). Prognostic role of pd-l1 expression in invasive breast cancer: A systematic review and meta-analysis. Cancers.

[48] Rozenblit M., Huang R., Danziger N., Hegde P., Alexander B., Ramkissoon S., Blenman K., Ross J.S., Rimm D.L., Pusztai L. (2020). Comparison of PD-L1 protein expression between primary tumors and metastatic lesions in triple negative breast cancers. J. Immunother. Cancer.

[49] Lotfinejad P., Kazemi T., Safaei S., Amini M., Baghbani E., Shotorbani S.S., Niaragh F.J., Derakhshani A., Shadbad M.A., Silvestris N. (2021). PD-L1 silencing inhibits triple-negative breast cancer development and upregulates T-cell-induced pro-inflammatory cytokines. Biomed. Pharmacother..

[50] Baandrup L., Sand F.L., Aalborg G.L., Nøttrup T.J., Fiehn A.M.K., Kjaer S.K. (2024). PD-L1 expression in vulvar cancer: A systematic review and meta-analysis. Histopathology.

[51] O’Meara T.A., Tolaney S.M. (2021). Tumor mutational burden as a predictor of immunotherapy response in breast cancer. Oncotarget.

[52] Vougiouklakis T., Vanderbilt C., Rana S., Mohanty A., Pareja F., Brogi E., Schwartz C., Arcila M.E., Ladanyi M., Wen H.Y. (2025). Genomic characterization of tumor mutational burden-high breast carcinomas. npj Precis. Oncol..

[53] Seino M., Sano S., Gonai Y., Horikawa S., Nakamura F., Okui Y., Matsukawa J., Sakaki H., Watanabe N., Yamauchi K. (2025). Investigation of tumor mutation burden using the comprehensive genomic profiling data of vulvar and vaginal malignant tumors: An observational study using C-CAT database. Int. J. Clin. Oncol..

[54] Corey L., Wallbillich J.J., Wu S., Farrell A., Hodges K., Xiu J., Nabhan C., Guastella A., Kheil M., Gogoi R.M. (2023). The Genomic Landscape of Vulvar Squamous Cell Carcinoma. Int. J. Gynecol. Pathol..

[55] Barroso-Sousa R., Pacífico J.P., Sammons S., Tolaney S.M. (2023). Tumor Mutational Burden in Breast Cancer: Current Evidence, Challenges, and Opportunities. Cancers.

[56] Choschzick M., Hoesli L., Stergiou C., Rüschoff J.H. (2026). Comprehensive genomic profiling of vulvar squamous cell carcinoma reveals subtype-specific mutational landscapes. Virchows Arch..

[57] Zach D., Åvall-Lundqvist E., Falconer H., Hellman K., Johansson H., Rådestad A.F. (2021). Patterns of recurrence and survival in vulvar cancer: A nationwide population-based study. Gynecol. Oncol..

[58] Woelber L., Mahner S., Voelker K., Zu Eulenburg C., Gieseking F., Choschzick M., Jaenicke F., Schwarz J. (2009). Clinicopathological prognostic factors and patterns of recurrence in vulvar cancer. Anticancer Res..

[59] Röhrmoser K., Ignatov A., Gerken M., Ortmann O., Klinkhammer-Schalke M., Papathemelis T. (2022). Risk factors and temporal patterns of recurrences in patients with vulvar cancer: Implications for follow-up intervals and duration. J. Cancer Res. Clin. Oncol..

[60] Yu B., Yan L., Wang H.Y., Yang J., Yang J. (2025). Invasive lobular carcinoma of the breast: Metastatic patterns and treatment modalities—A review. Front. Oncol..

[61] Khullar K., Patrich T., Jabbour S.K., Hathout L. (2022). Adjuvant Radiation in Early Stage Vulvar Cancer: A Review of Indications and Optimal Dose. Appl. Radiat. Oncol..

[62] Miljanović-špika I., Madunić M.D., Topolovec Z., Kenjereš D.K., Vidosavljević D. (2021). Prognostic Factors for Vulvar Cancer. Acta Clin. Croat..

[63] Liu P., Ji X., Li Z., Kong W., Pan Z., Deng M., Miao J. (2024). The prognostic significance of primary tumor site in vulvar cancer: A population-based cohort study. J. Gynecol. Oncol..

[64] Tessier-Cloutier B., Asleh-Aburaya K., Shah V., McCluggage W.G., Tinker A., Gilks C.B. (2017). Molecular subtyping of mammary-like adenocarcinoma of the vulva shows molecular similarity to breast carcinomas. Histopathology.

